# Application of Exercise Snacks across Youth, Adult and Clinical Populations: A Scoping Review

**DOI:** 10.1186/s40798-025-00829-6

**Published:** 2025-03-18

**Authors:** Kathryn L. Weston, Jonathan P. Little, Matthew Weston, Sara McCreary, Vanessa Kitchin, Amrit Gill, Ailsa Niven, Melitta A. McNarry, Kelly A. Mackintosh

**Affiliations:** 1 Department of Psychological Sciences and Health, University of Strathclyde, Glasgow, UK; 2 School of Health and Exercise Sciences, University of British Columbia, Okanagan, Kelowna, British Columbia Canada; 3 Applied Sports, Technology, Exercise and Medicine (A-STEM) Research Centre, Swansea University, Swansea, UK; 4 Institute for Sport, Physical Education and Health Science, Moray House School of Education and Sport, University of Edinburgh, Edinburgh, UK; 5 Institute of Sport, Manchester Metropolitan University, Manchester, UK; 6 Physical Activity for Health Research Centre (PAHRC), University of Edinburgh, Edinburgh, UK

## Abstract

**Background:**

Interest in ‘exercise snacks’ has increased, yet a comprehensive and holistic review of this novel concept is lacking. We aimed to map global research on ‘exercise snacks’, across youth, adult and clinical populations through a scoping review.

**Methods:**

A systematic search was conducted in six databases. Grey literature searches were also conducted. Studies whereby participants were prescribed a structured bout of intense exercise dispersed across the day, or the exercise was explicitly defined as a form of ‘snacks’, in any setting were included. We used the Consensus on Exercise Reporting Template (CERT) to assess the completeness of exercise descriptions. Data were recorded into spreadsheets, then descriptively analyzed and summarized in graphic form.

**Results:**

The 45 publications meeting our inclusion criteria represented 33 original studies. These 33 studies enrolled a total of 1118 participants, with a median sample size of 24. Studies were categorized as either acute (*n* = 12) or chronic (*n* = 21) trials with both trial types performed across a wide range of participant ages (range 8.7 to 78 years) but mostly conducted on healthy adults and older adults. The majority of studies (20/33) defined the concept as ‘exercise snacks’, with study context being predominantly the laboratory or home. A wide variety of exercise modes (e.g., cycling, stair climbing, body weight exercises) and comparator conditions (e.g., moderate intensity continuous exercise, prolonged sitting, non-exercise controls) were used. ‘Exercise snack’ intensity was prescribed more frequently than it was reported, and, of the available data, mean intensity was estimated at 76.9% of maximal heart rate and 5.2 Arbitrary Units (AU) on the Ratings of Perceived Exertion (RPE) CR10 scale. Study outcome measures were predominantly cardiovascular, metabolic, muscular, and psychological, with studies mostly adhering to the CERT, though there was underreporting of detail for the exercise provider, motivation strategies, adverse events and intervention fidelity.

**Conclusion:**

The ‘exercise snack’ concept is being increasingly used to cover an array of exercise models. The most common protocols to date utilize body weight exercises or stair climbing. We recommend ‘exercise snacks’ terminology is consistently used to describe protocols whereby short, purposeful structured exercise is dispersed throughout the day. Future studies should provide detailed descriptions of their ‘exercise snacks’ model, through exercise and adverse event reporting checklists.

**Supplementary Information:**

The online version contains supplementary material available at 10.1186/s40798-025-00829-6.

## Key Points

• A total of 1118 participants across 33 original studies resulting in 45 publications met our inclusion criteria, examining the application of intense exercise bouts dispersed across the day (i.e., ‘exercise snacks’).

• Many types of exercise (including cycling, stair climbing, and body weight exercises) were used in ‘exercise snacks’ studies across a wide age range of participants, with most trials performed in the laboratory or at home.

• Researchers and practitioners should ensure consistent use of the term ‘exercise snacks’ for describing protocols where short, purposeful structured exercise is dispersed throughout the day and provide detailed descriptions of how their exercise prescription is designed and completed by participants.

**Supplementary Information:**

The online version contains supplementary material available at 10.1186/s40798-025-00829-6.

## Background

Across the human lifespan, positive relationships between physical activity, cardiorespiratory and muscular fitness, and health are well established [1–5]. Moreover, physical inactivity, wherein physical activity levels do not meet the current physical activity guidelines [6], is a long known major contributor to cardiovascular disease risk [7]. Distinct from physical inactivity, sedentary behavior, defined as any waking behavior characterized by an energy expenditure < 1.5 metabolic equivalent of task [MET]), while in a sitting, lying or reclining position [6], can have negative health consequences beyond those associated with low levels of physical activity [6, 8]. The recognition of these interrelated influences on health has led to guidance around ‘sitting less and moving more’ [7], which has been operationalized in various ways, including the promotion and prescription of physical activity and exercise, respectively.

Despite public-health messaging on the benefits of moving more [9], perceived lack of time represents a major barrier to exercise participation in various population subgroups and contexts [10]. As such, health-enhancing time-efficient alternatives to traditional aerobic exercise (i.e., continuous moderate-intensity training) are potentially attractive. Examples include sprint-interval training (SIT) and high-intensity interval training (HIIT), which are broadly defined as brief bouts of intense exercise interspersed with recovery [11]. Both have demonstrated efficacy for improving various cardiovascular and metabolic risk factors in youth [12], healthy adults and some clinical populations [11, 13, 14], with improvements largely consistent with traditional aerobic training [15, 16]. This body of literature, along with emerging evidence that activity bouts of any duration are associated with improved health outcomes [17, 18] are now reflected in the most recent World Health Organization [19], United States of America [20] and United Kingdom (UK) [21] Physical Activity Guidelines for adults, where the potential health benefits for shorter bouts of vigorous activity (i.e., < 10 min) accumulated over the day are acknowledged. The UK physical activity guidelines also highlight the potential for even shorter durations of very vigorous intensity activity (e.g., sprinting or stair climbing) to confer health benefits.

Despite the associated benefits of an active lifestyle, humans often exist in environments that may not only limit their physical activity and exercise opportunities but also promote sitting for prolonged time periods [8, 22]. This highlights the need to investigate novel means of accumulating time-efficient physical activity, which can enhance health and/or partly offset the deleterious consequences of sedentary behavior. One such approach is ‘exercise snacks’, which we define as a structured bout of intense exercise dispersed across the day, with the quotation marks removed herein to enhance readability. The concept of snacks within physical activity and exercise is gaining increased interest; however, there is a lack of definitional consistency and nuanced differences between different snack concepts.

One type of exercise snack is sprint snacks, which have previously been defined as isolated ≤ 1-minute bouts of vigorous-intensity exercise performed periodically throughout the day [23, 24]. Here, each sprint is performed several hours apart, on the basis that performing several snacks within the same day could potentially make SIT more appealing, even more time efficient, and potentially easier to implement [23]. Findings from a recent narrative review suggest that this type of exercise snacking shows promise as a novel strategy for improving cardiometabolic health in adults [25], with the caveat that the current evidence base is formed predominantly of laboratory-based proof-of-concept trials with small sample sizes. Other researchers have used the term strength snacks as a way of describing low-dose resistance training [26, 27].

The concept of exercise snacks has similarities with the novel physical activity promotion strategies of Vigorous Intermittent Lifestyle Physical Activity (VILPA; e.g [28])., and Snacktivity™ [29] (i.e., brief or bite size bouts of incidental physical activity performed sporadically during activities of daily living) but can be differentiated. Physical activity is broadly defined as bodily movement produced by skeletal muscles requiring energy expenditure [19] with VILPA and Snacktivity™ focusing on a variety of unstructured and incidental lifestyle physical activities performed across the day. Exercise, however, represents a subset of leisure-domain physical activity as the exercise is intentional, structured, and repetitive [19, 25, 30], thus enabling alignment to the core exercise training program principles of frequency, intensity, time, type, volume, and progression (FITT-VP) [31]. Based on these definitions, exercise snacks, as structured bouts of intense exercise dispersed across the day, fall under the category of exercise, not general lifestyle physical activity. This distinction between incidental unstructured vs. intentional structured activity is also important when considering promotion of each behavior, as the psychological determinants of behavior change likely differ [32, 33].

In addition to the purposeful/intentional nature, bout intensity may also distinguish exercise snacks from VILPA and Snacktivity™. First, however, it must be acknowledged that characterizing and quantifying the intensity of brief, isolated exercise bouts is challenging [25]. This is due to the oxygen deficit incurred at the onset of exercise, whereby the time required for ventilation, heart rate and oxygen consumption to increase in proportion to the exerted mechanical power [34] and attain a workload equilibrium [35] may be longer than the bout itself. Consequently, heart rate is typically not the best indicator of intensity for supramaximal exercise or submaximal high-intensity short-duration intervals (< 30 s; [36]). Ratings of perceived exertion are strongly and positively associated with breathing frequency [37] and the volume of exercise completed [38], which may not be conducive for accurately characterizing an intense, yet short, bout of exercise performed within an exercise snack. As such, traditional metrics of exercise quantification may underestimate bout intensity and not differentiate exercise snacks from VILPA and Snacktivity™. From an intensity perspective, what most likely separates the concepts of exercise snacks, VILPA and Snacktivity™, is the intensity used to prescribe, not quantify, bout intensity. For example, the prescribed effort or absolute intensity of exercise snacks (e.g., “all-out” [23] and “as quickly as possible” [24, 39] is likely greater than the intensities associated with many moderate-to-vigorous incidental daily life activities, such as doing housework, marching on the spot and walking meetings at work [29] or brisk walking and playing with children [28].

Despite the nascent nature of the exercise snacks research field, four topical reviews have already been published [25, 40–42]. While all four make some contribution to the overall exercise snacks knowledge base, each contains distinct limitations and/or have omitted key studies. The first narrative review largely focused on the efficacy of sprint-type exercise snacks [25]. The second discussed the potential of exercise snacks for those living with and beyond cancer but did not present any data directly related to exercise snacking in this clinical population [40]. The third was a scoping review that combined the concepts and effects of exercise snacks, VILPA and Snacktivity™ in adults and older adults [41], despite the likelihood that each model could give rise to different physiological and psychological responses and should therefore be synthesized separately. Further, while Jones et al. [41] recommend detailed information about exercise snack interventions should be described using established intervention reporting guidelines [43, 44], these processes were not conducted in their review. Also, as their review focus was limited to adults and older adults, the use of the exercise snacks concept in children and adolescents remains unknown. Finally, the narrative review by Wang et al. [42] only incorporated data from a limited number of studies on sedentary adults, and mainly focused on potential mechanisms subtending the physiological responses observed to date. It is also pertinent to note that none of the four published reviews to date have acknowledged literature around strength snacks [26], or those which have utilized principles of exercise snacking but for the primary purpose of breaking-up sedentary time, rather than the promotion of exercise per se (e.g [45]). Therefore, there remains a dearth of knowledge to facilitate a holistic and comprehensive review and summary of this exciting new concept. These shortcomings highlight the need for a more comprehensive scoping review that maps and appraises all the emerging evidence relating to exercise snacks, in terms of study population, study concept and design, exercise prescription model, context and outcome measures of interest. Indeed, scoping reviews are a type of evidence synthesis appropriate for systematically summarizing a field of research not yet comprehensively reviewed [46], answering broad research questions (i.e., what is known about a particular concept [47]), and, therefore, a useful approach for examining emerging evidence [48].

### Aim and Objectives

The aim of this scoping review was to map the current state of exercise snack research. To do this, we addressed the following research questions:

1. What populations, concepts and contexts are targeted in exercise snack research?
2. What are the research limitations and gaps?
3. What recommendations should be provided to those involved in the science and practice of exercise snacks prescription and promotion?

## Methods

Our scoping review conforms to the guidelines set out by Levac and colleagues [49], The Joanna Briggs Institute (JBI) [50] and The Preferred Reporting Items for Systematic Reviews and Meta-Analyses-Scoping Review Extension [47] (Supplementary File 1). Our protocol was registered on the Open Science Framework (http://osf.io/h8xrs).

### Eligibility Criteria

As the aim of our scoping review was to map the available evidence on the application of exercise snacks, we placed as few eligibility restrictions as possible and aligned our criteria to the PCC (Population, Concept [including Outcome], and Context) approach recommended by Peters et al. [46]. Eligible studies included human participants of any age (population) who were prescribed exercise snacks that were dispersed across the day (concept) in any setting (context). To ensure a balanced and complete overview of the available evidence [51], we included grey literature in our systematic searches (Table 1).

**Table 1:** Scoping review eligibility criteria based on study population, concept, outcome, context and publication types

	Inclusion	Exclusion
Population	- Human studies - Participants of any age - Participants of any sex - Participants of any health status (e.g., healthy, unhealthy, clinical)	- Animal studies
Concept	- Original studies, whereby participants were prescribed a structured bout of intense exercise (i.e., defined as “all-out” [23] or “high-intensity”, or performed “as quickly as possible” [24, 39], or included an objective measure of intensity which indicated prescribed exercise was of at least a vigorous intensity [e.g., ≥ 80% maximal heart rate [52]]) dispersed across the day OR the exercise is explicitly defined by the authors as “snacks”, e.g., “exercise snacks”, “strength snacks”, “sprint snacks”.	- Studies involving unstructured, short sporadic bouts of low, moderate, and vigorous incidental lifestyle physical activities (e.g., carrying shopping, playing with children) -Studies involving a single, isolated bout of exercise (i.e., not dispersed or fragmented) - Studies involving structured bouts of physical activity prescribed as low or moderate intensity, accumulated or dispersed across the day
Outcomes	- Cardiovascular (directly or indirectly assessed) - Muscular (e.g., strength or power) - Respiratory - Vascular (e.g., hemodynamics) - Metabolic (e.g., glucose tolerance, insulin resistance, lipid profiles) - Inflammatory - Anthropometric (e.g., measures beyond height, body mass, body mass index, such as skinfolds or muscle mass) - Psychological (e.g., affective responses, quality of life) − 24-hour movement behavior (e.g., sleep, sedentary behavior, physical activity) - Appetite	- None
Context	- Any research setting where the concept has been prescribed	- None
Publication Type	- All research study designs including any methodology (e.g., qualitative, quantitative, and mixed methods) - Original academic journal papers, grey literature and brief reports focusing on Sport and Exercise/ Health Science - Articles written in English - All years from 1946 to 2024	- Editorials, opinion pieces, magazine/ newspaper articles, and papers without primary data - Systematic, scoping, or narrative reviews (individual studies from any such reviews included if relevant) - Study protocols (e.g., trial registries)

### Information Sources and Search

A preliminary search of four databases (CINAHL, MEDLINE, SPORTDiscus, Web of Science) was undertaken by one of the authors (MW), with the search terms informed by previous systematic and scoping reviews [18, 28]. Analysis of text words contained in the title and abstract, and of the index terms used to describe the article was then undertaken [46]. Following this, a refined search strategy, a critical step in scoping review methodology [49], was drafted by a medical librarian (VK) and further refined through discussion among the multidisciplinary research team. Following team agreement, our systematic search strategy was subjected to a formal peer review using the Peer Review of Electronic Search Strategies (PRESS) checklist to identify errors and potential enhancements in the selection of subject headings and text words [52]. This process involved VK submitting the refined Ovid MEDLINE search strategy for blind peer review by another health sciences librarian experienced in systematic searches who suggested that how the concepts were grouped, certain search terms were articulated and what proximities were used were reconsidered. Consequently, we devised our search sets in MEDLINE using three main concept blocks (intensity, bouts/intervals, and exercise) and searches were performed by our specialized research librarian (VK). We created strategies in MEDLINE and Embase on Ovid and adapted them to CINAHL (Ebsco), Web of Science, Scopus, and SPORTDiscus (Ebsco).

A systematic search of grey literature is considered important for establishing a comprehensive and balanced view of the available evidence [51, 53]; therefore, we searched ProQuest Dissertations and Theses Global and PolicyCommons for relevant theses, dissertations, and conference proceedings. Our search strategies for all databases are available in Supplementary File 2, and in our open access protocol, located at http://osf.io/h8xrs.

We also performed manual searches of the conference proceedings from the American College of Sports Medicine (ACSM), European College of Sport Science (ECSS), and the British Association of Sport and Exercise Sciences (BASES). Reference lists of included articles were manually searched for potential studies not yet identified [47]. Prior to manuscript submission, identified articles were reviewed to ensure that no study had been retracted between inclusion and publication. The final search date was 27th November 2024.

### Selection of Sources of Evidence

Database searches were exported into Covidence systematic review software (Veritas Health Innovation, Melbourne, Australia, available at www.covidence.org) for screening. Duplicate studies were automatically removed. To assure quality control in screening, title and abstracts of all studies were independently screened by three authors (AG, SM & KW) to gauge shared understanding of the selection criteria, discuss any disagreements and further specify the inclusion and exclusion parameters [53]. Papers that failed to meet our Population, Concept, and Context were excluded at this stage of the selection process. Following this, full texts of remaining articles were independently screened by two authors (SM, KW). After full-text screening, those studies recommended for exclusion were reviewed by a third author (MW) to confirm consistency in exclusion criteria application.

### Data Extraction

Using the guidance provided by Aromataris et al. [54] for scoping review data extraction, an initial data extraction form was created. This was modified to fully accommodate our review question, along with population, concept, and context [55] and two authors (KW, MW) trialed the extraction form [50]. As scoping reviews synthesize and describe evidence coverage, a risk of bias assessment across individual studies is not applicable [47, 55]. Table 2 details the variables that were extracted from the included studies.

**Table 2:** Data extraction variables

Sub-category	Data collected
Study details	Author (s); Full reference; Study ID; Publication year; Country of origin; Report registered (yes, no); Study aim/aims (as reported by study authors); Study design; Pilot study (yes, no); Study type (acute, chronic).
Population	Study population; Age (years); Participant ethnicity reported (yes, no); Population femaleness (ratio of female participants to male participants); Menstrual cycle phase reported (yes, no, not relevant)
Concept	Exercise defined by authors as “snacks” (yes, no); Exercise as described by authors; Exercise mode; Exercise sample size (n); Exercise program length; Exercise weekly frequency; Exercise intensity (yes, no); Author prescribed intervention intensity (e.g., 90% maximal heart rate); Exercise intensity measurement (e.g., heart rate, ratings of perceived exertion); Exercise intensity reported (yes, no); Method of intervention intensity confirmation (e.g., means, within-subject standard deviations); Reported actual intervention intensity; Intervention warm-up (yes, no); Exercise snack bout duration (s); Number of daily exercise snack bouts; Total daily exercise snack duration (s); Time between exercise snacks; Intervention as replacement or in addition to usual activity; Comparator; Comparator sample size
Outcomes	Study outcomes
Context	Study location (e.g., school, home, laboratory)
Analysis	*a*-priori power calculation (yes, no); Statistical inferential approach (i.e., null hypothesis significance testing, effect sizes); Between-group comparisons (yes, no).
Findings	Key study findings (as reported by study authors)

We used the Consensus on Exercise Reporting Template (CERT) [44] to assess the completeness of exercise descriptions. This checklist is an extension of item 11 of the Template for Intervention Description and Replication (TIDieR) checklist [43] and comprises 16 items that represent the minimum recommendation for describing an exercise intervention.

### Synthesis and Presentation of Results

Data from the included studies were extracted by two authors (MW and KW) in duplicate, then checked by two authors (JL and AN). Data was extracted into two Microsoft Excel spreadsheets. The first sheet contained individual study-level data, and the second sheet contained the CERT results. Data from both sheets were descriptively analyzed by quantifying text and frequency counts of data extraction items [55] with summaries of study populations, settings, methods, and outcomes all visualized in graphic form. Descriptive analyses and visualizations [56–58] were performed in R (version 4.1.2, R Foundation for Statistical Computing) using the *dplyr*, *tidyverse, ggplot2, lemon, and UpsetR* packages with the PRISMA flowchart created via the Shiny app [56, 59]. These packages enabled creative ways of conveying our results to those involved in the science and practice of exercise snack prescription and promotion, and avoided the use of large tables that can be difficult to read in a standard journal article format [55]. Our data extraction sheet containing the raw data can be found in Supplementary File 3 and is available open access at http://osf.io/h8xrs.

In line with JBI scoping review guidance, we make no attempt to draw conclusions regarding the efficacy and/or effectiveness of exercise snacks for any population or outcome in the presentation and interpretation of our results. This is due to the included studies not having undergone a process of critical appraisal/risk of bias appraisal and, also, not having undergone a process of pooling or aggregation that considers the combination of all study results (e.g., meta-analysis or meta-synthesis) [55].

## Results

### Study Selection

**Fig. 1:**
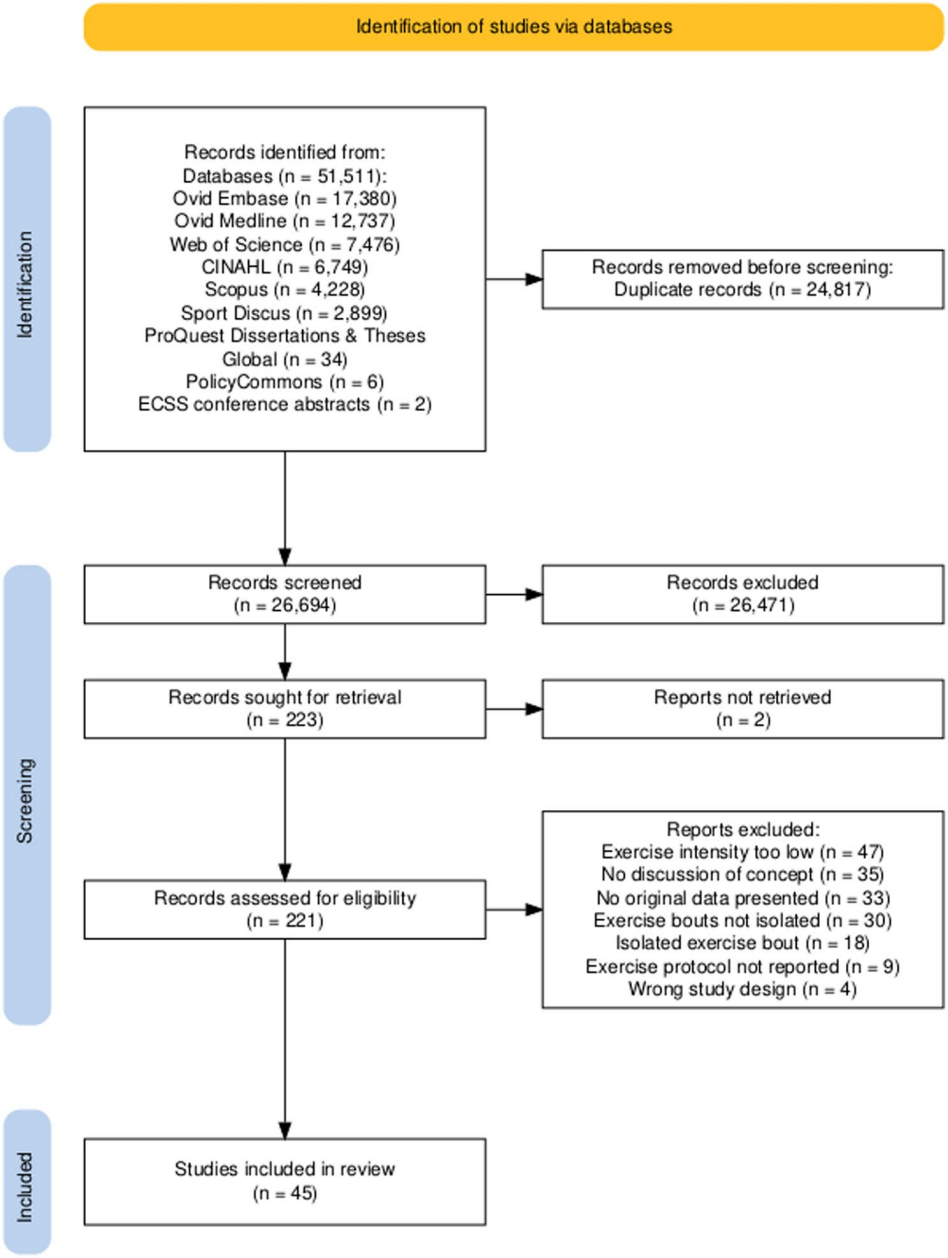
PRISMA flow chart outlining study inclusion process

**Fig. 2:**
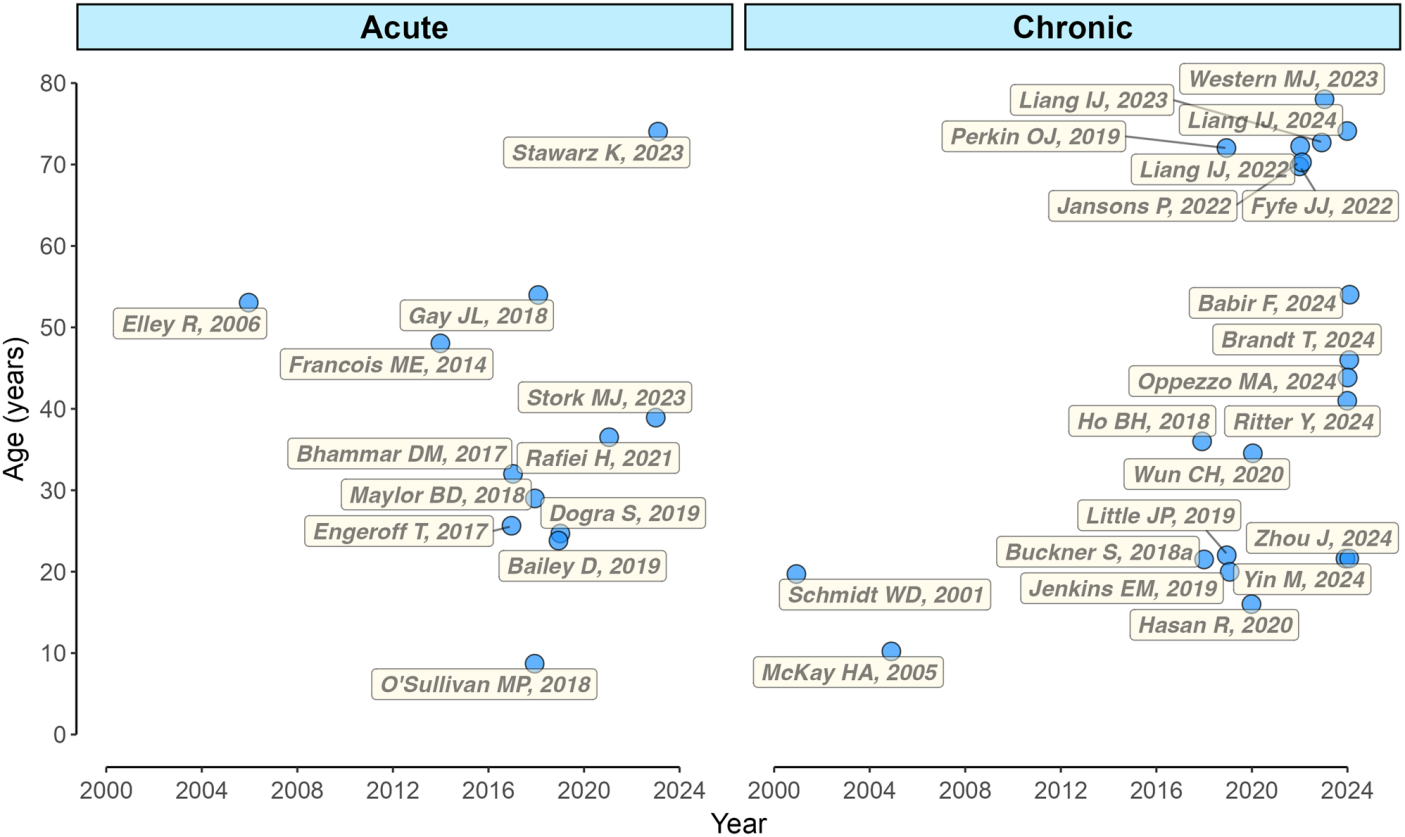
Publication year and participant mean age for the 33 original studies in our scoping review

Following the database searches, 51,511 records were identified (Fig. 1). Once duplicates were removed (*n* = 24,817), 26,694 titles and abstracts were screened for inclusion, resulting in 223 full-text articles being sought for retrieval. Two records were not retrievable; therefore 221 full-text articles were screened for eligibility. Subsequently, 176 publications were excluded and 45 were included. These 45 publications [23, 24, 26, 27, 39, 45, 60–98] came from 33 original studies (i.e., some studies resulted in multiple publications). For example, Nagy et al. [65], Ajibewa et al. [66], Block et al., [72], Weston et al. [75], and Nagy et al. [77] are all from one registered study (clinical trials identifier NCT02831309), whereby the primary outcome was exercise energy expenditure [67]. As scoping review guidance is to make efforts to avoid counting the same data items multiple times from different sources [55], the original 33 studies represent our unit of synthesis. The earliest published study was in 2001 (Fig. 2), with a notable increase in publication activity from 2017 (82% of studies included in the review). The number of original studies per country was eight (UK), seven (Canada), seven (USA), three (Germany), two (Singapore), two (Australia), two (New Zealand), and two (China).

### Study Characteristics

Study designs were randomized controlled trials (*n* = 12), randomized crossover trials (*n* = 10), single group pre-post trials (*n* = 4), non-randomized controlled trials (*n* = 2), a qualitative study (*n* = 1), a non-randomized cross-over trial (*n* = 1), a prospective cohort trial (*n* = 1), a matched-controlled cohort trial (*n* = 1), and a user-centered design process study (*n* = 1). These study designs have subsequently been grouped as Acute (i.e., single exercise sessions/ conditions [*n* = 12]) or Chronic (i.e., repeated exercise sessions/ exercise training studies [*n* = 21]). Of the 33 original studies, 40% were registered on trial registries, 52% were described as pilot or feasibility studies, and 33% provided an *a*-priori sample size estimation.

### Population

**Fig. 3:**
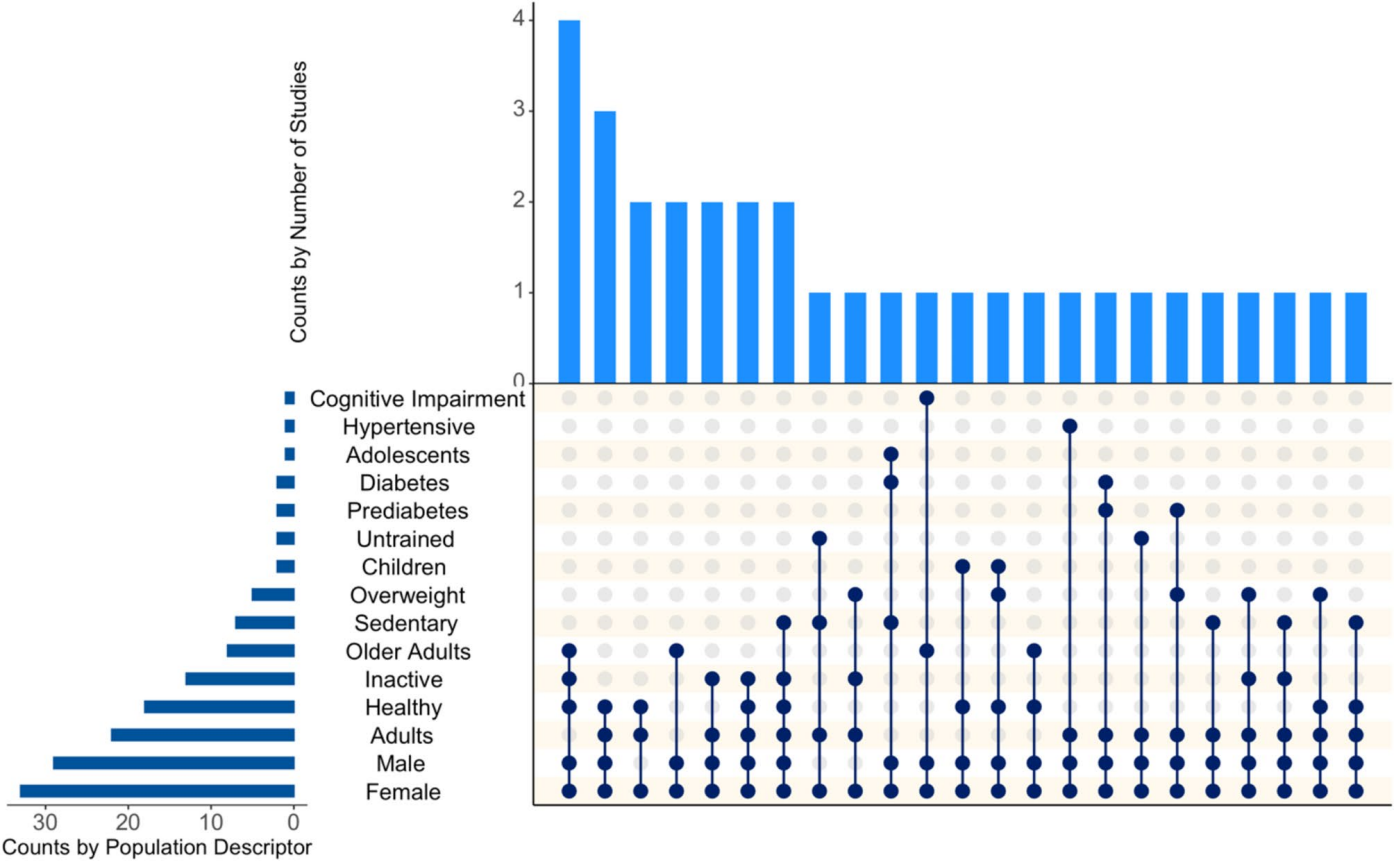
Upset plot showing the frequency and different combinations of populations and population descriptors across the 33 exercise snacks studies

A total of 1118 participants were enrolled into the 33 studies with 1035 participants completing the studies, giving a median study sample size of 24 participants (range 8 to 124 participants). Of the 1035 participants, 830 (median 33, range 8 to 124) participants were involved in the Chronic studies and 205 (median 14, range 9 to 39) participants in the Acute studies. All 33 studies involved female participants, with 29/33 involving male participants and no studies including only males. Studies represented a broad age range (median 36.5 years, range 8.7 to 78 years), with the majority conducted in adults and older adults (Figs. 2 and 3). Three studies included children or adolescents aged < 18 years, 27% of studies described participant ethnicity, and 25% studies provided details of female participant menstrual cycle, where relevant (i.e., the median participant age was ≥ 10 years and ≤ 45 years [99, 100]. Beyond sex and age, there was a broad range of population descriptors (*n* = 9) with the most popular being healthy (18/33 studies), inactive (*n* = 13), and sedentary (*n* = 7; Fig. 3).

### Concept, Context and Exercise Mode

**Fig. 4:**
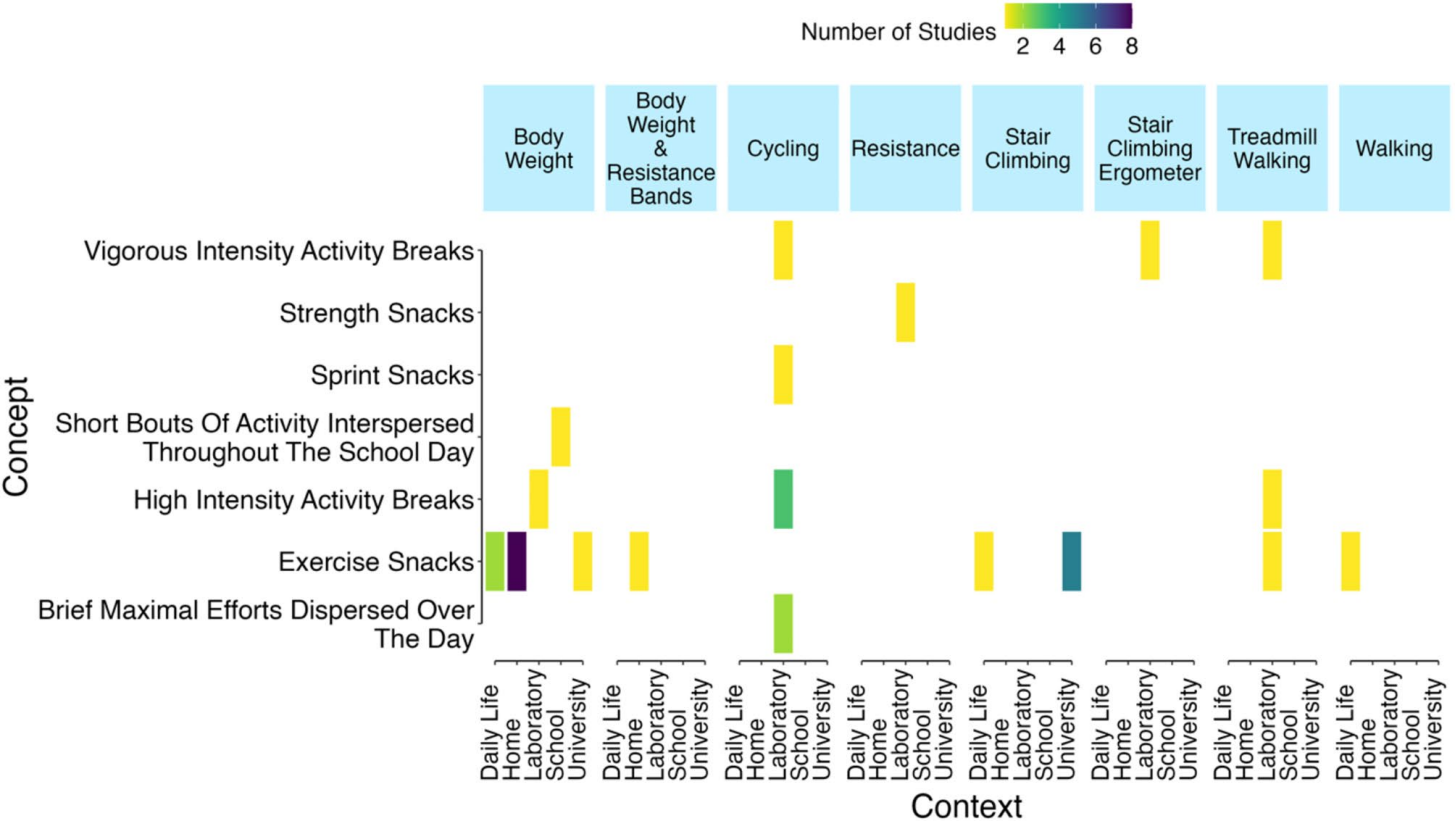
Frequency of study concept (i.e., exercise definition) by context (i.e., location) grouped by exercise mode

Concept was described by study authors in seven different ways (Fig. 4), with exercise snacks being the most frequent exercise description (20/33 studies). Context was distributed across five locations (laboratory [*n* = 13], home [*n* = 9], university [*n* = 6], daily life [*n* = 4], school [*n* = 1]) using eight exercise modes (body weight [*n* = 13], body weight and resistance bands [*n* = 1], cycling [*n* = 7], resistance [*n* = 1], stair climbing [*n* = 6], stair climbing ergometer [*n* = 1], treadmill walking [*n* = 3], walking [*n* = 1]). The most popular combination of concept, context, and mode was exercise snacks performed at home using body weight (*n* = 8).

### Exercise Prescription Characteristics

**Fig. 5:**
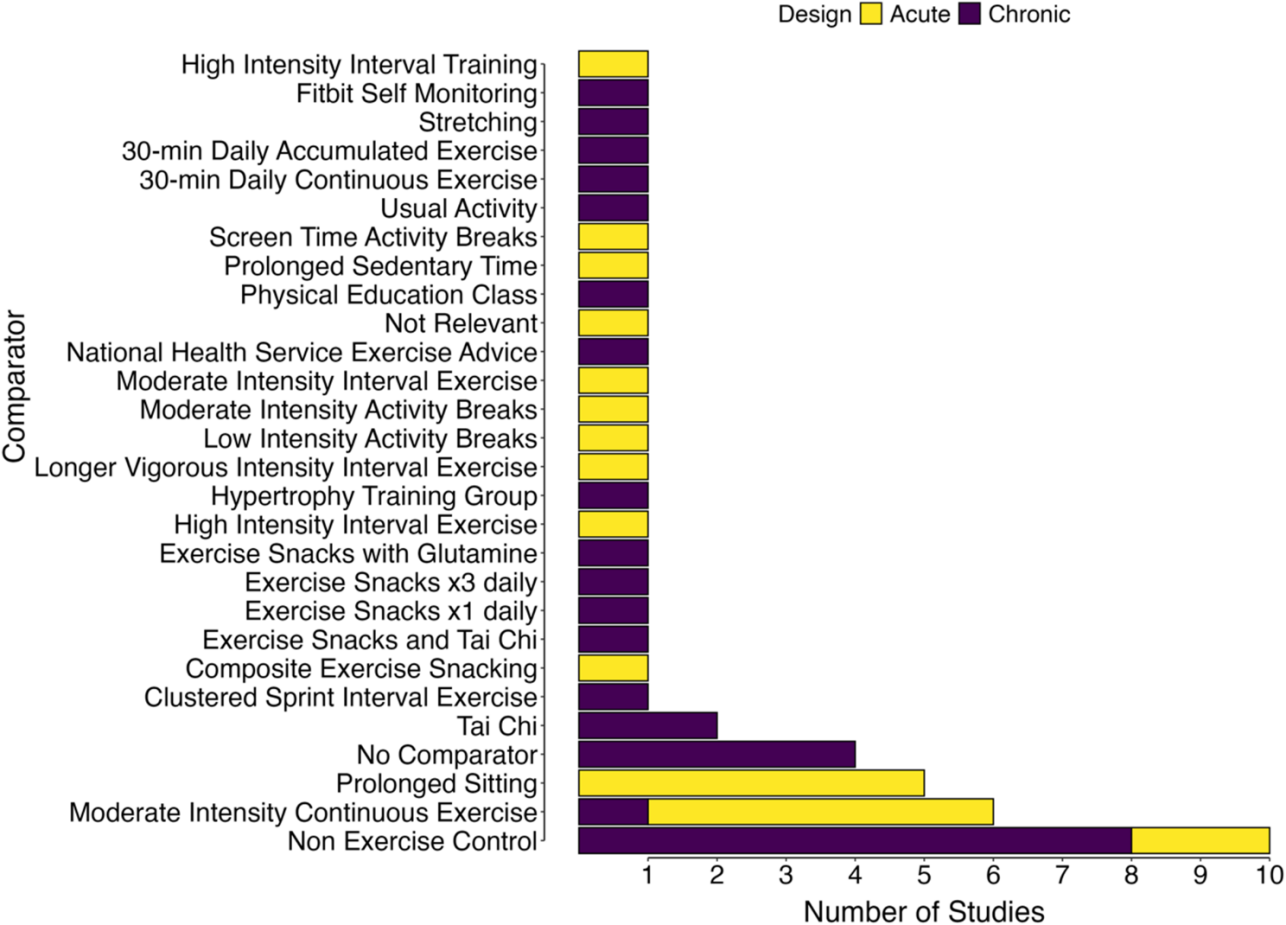
Bar chart of study comparators colored by study design

A wide variety of comparators (*n* = 28) were used (Fig. 5) with the most frequent being non-exercise controls (*n* = 10), moderate intensity continuous exercise (*n* = 6), and prolonged sitting (*n* = 5). Of the 21 Chronic trials, eight had a non-exercise control and four had no comparator.

A pre-exercise warm-up was reported in 40% of studies. For the Chronic studies, median exercise snack intervention duration was 7 weeks (range 4.0 to 34.8 weeks), with snacks performed on a median of 4 days per week (range 2 to 7 days). Across all 33 studies, median exercise snack duration was 120 s (range 20 s to 720 s), median number of exercise snacks per day was 3 (range 1 to 20), median daily exercise snack duration was 960 s (16 min; range 60 s to 2,400 s) and the median rest between exercise snacks was 60 min (range 1.5 min to 240 min).

**Fig. 6:**
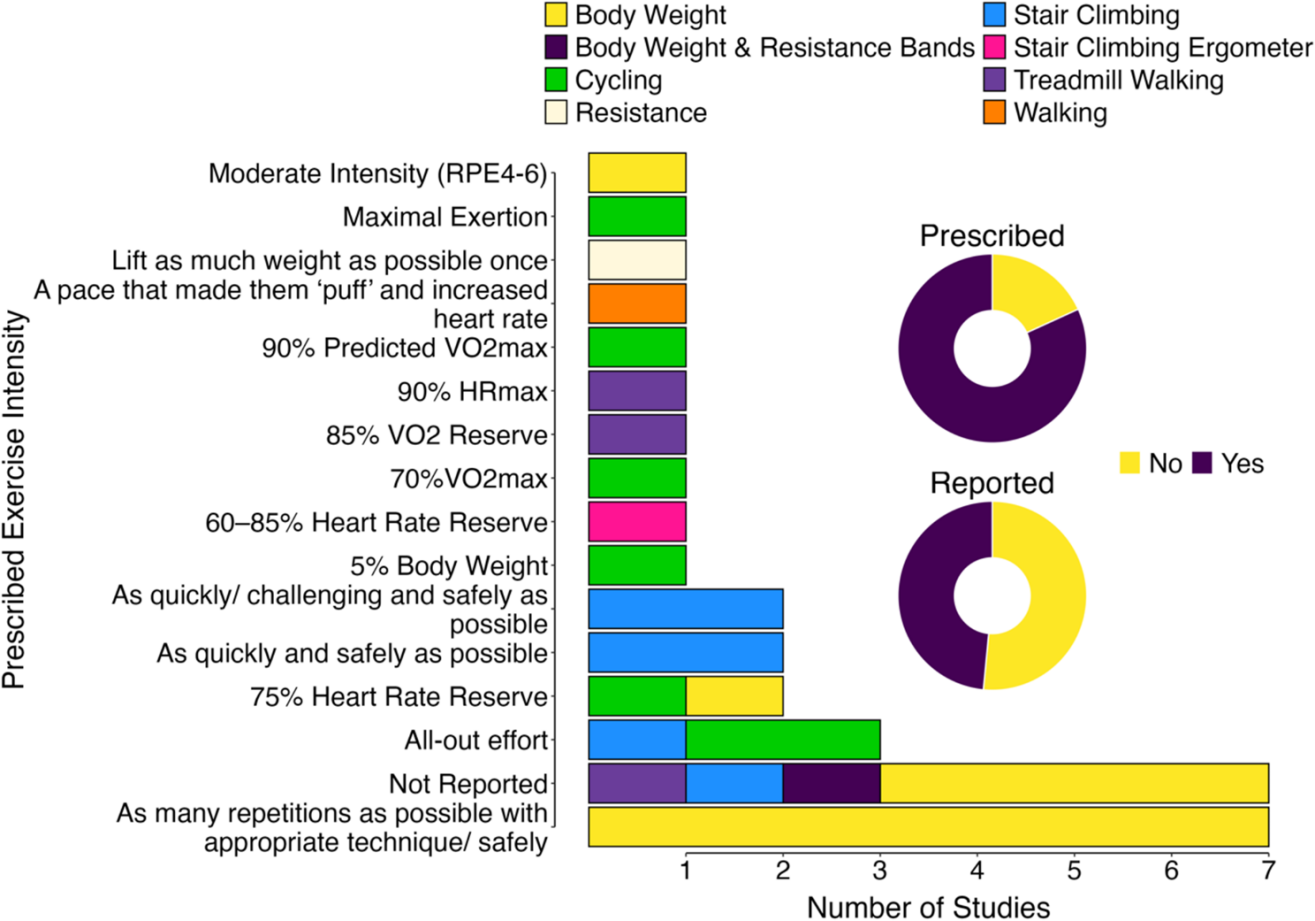
Bar chart showing the different ways of exercise prescription, grouped by exercise mode, along with the proportion of studies reporting a prescribed exercise intensity and reported exercise intensity (inset)

Most studies (82%) prescribed exercise intensity (Fig. 6, inset), with the most frequent being ‘as many repetitions as possible with appropriate technique/ safely’ (*n* = 7; Fig. 6) or non-reporting (*n* = 7). Prescribed intensity was not reported in 10/33 studies. Exercise intensity was reported in 49% of studies (Fig. 6, inset). The most prevalent exercise intensity measures were heart rate (15% of studies), RPE (CR10 scale; 12%), and heart rate combined with RPE (CR10 scale; 12%). Other measures of exercise intensity were treadmill speed, power, power combined with RPE (CR10 scale; 6–20 scale), heart rate combined with RPE (6–20 scale), resistance (expressed as kilograms), percentage of maximal oxygen consumption (%VO_2max_, power associated with %VO_2max_), and RPE (OMNI resistance scale). Ten studies did not report how exercise intensity was measured but in one of these studies the maximum number of steps climbed during exercise was reported [91]. The mean ± SD of exercise intensity measures was the sole method of exercise intensity confirmation (16/33 studies).

**Fig. 7:**
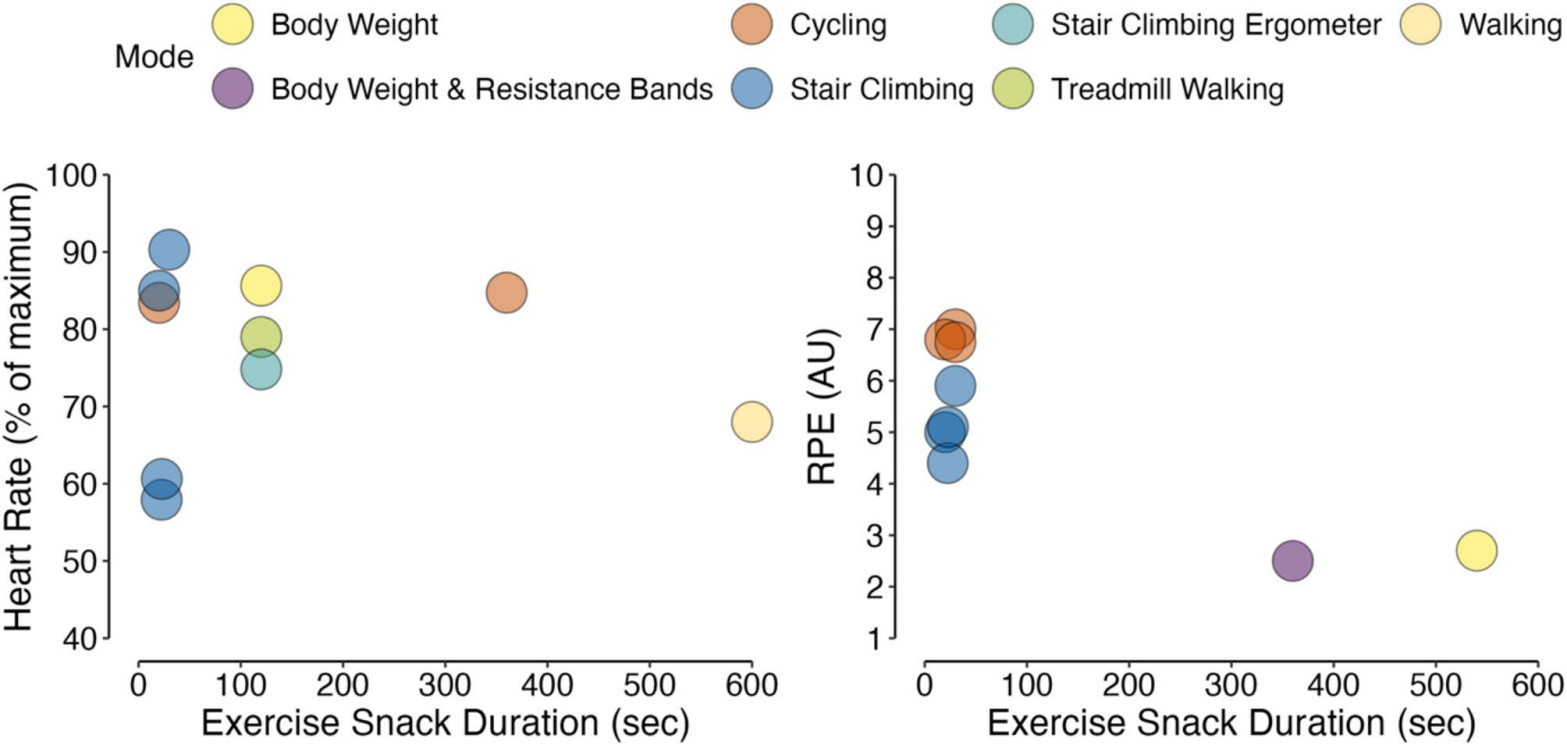
Mean study exercise snack heart rate (*n* = 10) and RPE (*n* = 9*) colored by exercise mode and plotted against exercise snack duration.* Reference [96] reported exercise intensity via RPE (~ 6.0 AU) but provided no precise exercise snack duration

In an attempt to reconcile as much exercise snack intensity data as possible, we took heart rate data from five studies [23, 39, 64, 67, 98] and converted this into a mean exercise snack heart rate expressed as a percentage of maximal heart rate (%HRmax), using a generalized equation for predicting maximal heart rate [101], and also converted OMNI Resistance RPE [79] and 6–20 RPE [70] to the corresponding qualitative descriptor on the CR10 scale. Consequently, mean exercise snack heart rate and RPE (CR10) was 76.9 ± 11.2% HRmax (*n* = 10 studies) and 5.2 ± 1.6 AU (*n* = 10 studies), respectively (Fig. 7) with the mean RPE equating to a perceived exertion of ‘hard’ on the CR10 scale.

### Consensus of Exercise Reporting Template (CERT)

**Fig. 8:**
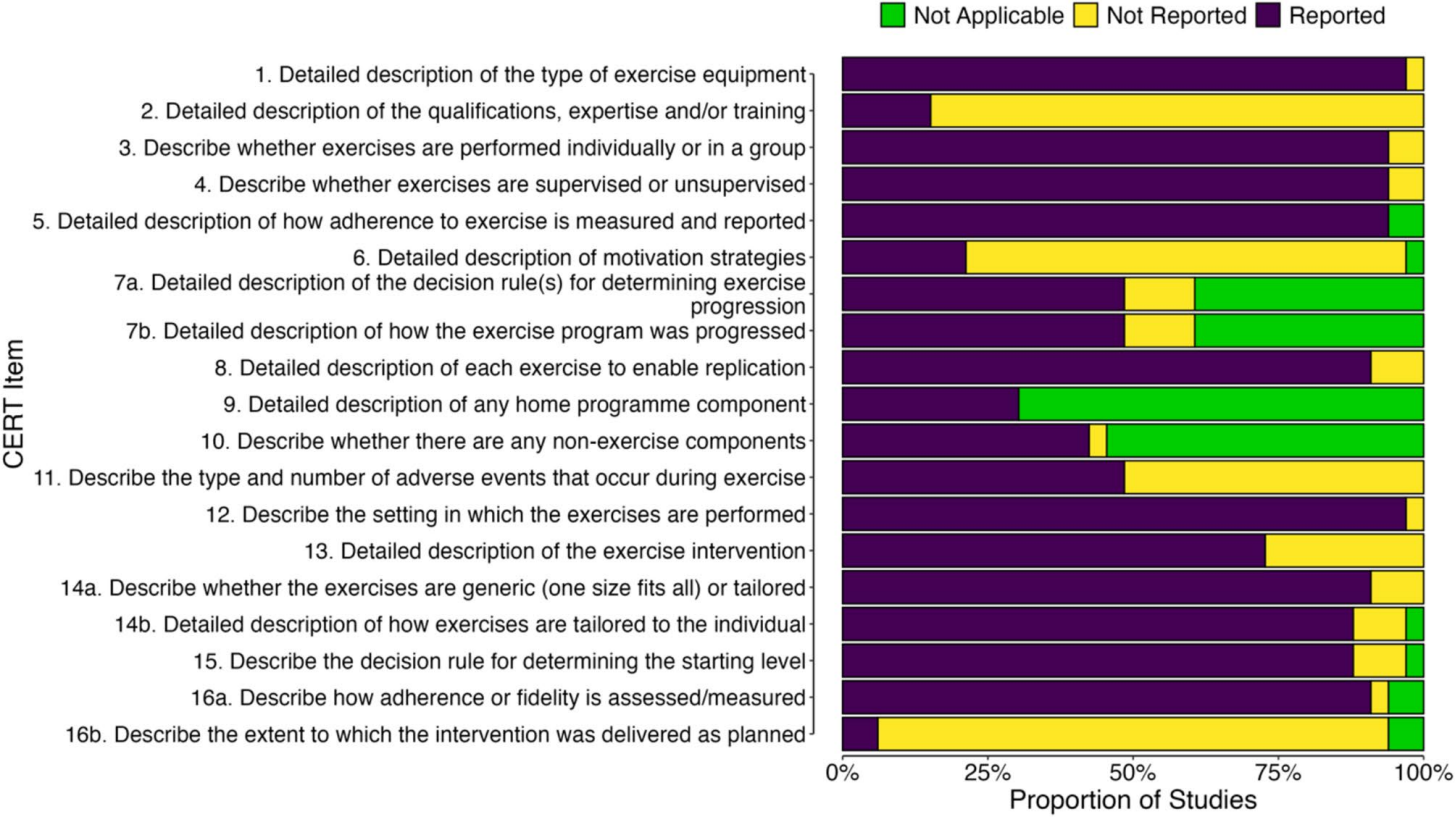
Proportion of reported, not reported and not applicable items for each study when evaluated against the 16 CERT items

For most CERT reporting items, studies adhered to the reporting template, although four items were consistently underreported: Item 2 (qualification of the exercise provider: 5/33 studies), Item 6 (motivation strategies: 7/33 studies), Item 11 (adverse events: 16/33 studies), and Item 16b (intervention fidelity: 2/33 studies; Fig. 8).

### Study Outcomes

**Fig. 9:**
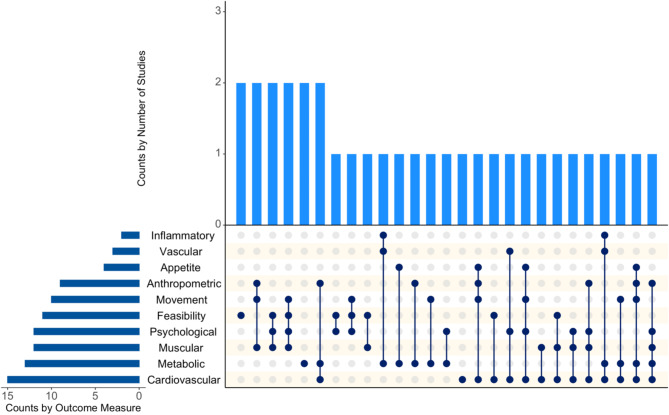
Upset plot showing the frequency and combination study outcomes

A wide variety (*n* = 87) of distinct physiological, psychological and behavioral outcome categories were utilized across the 33 studies with the most frequent categorization of outcomes being cardiovascular (e.g., cardiorespiratory fitness, blood pressure etc.: *n* = 15), metabolic (e.g., blood glucose, lipid metabolism etc.; *n* = 13), muscular (e.g., power: *n* = 12), and psychological measures (e.g., quality of life, enjoyment etc.: *n* = 12; Fig. 9). For full details on individual outcome measures from included studies, please see Supplementary File 3 and/or our open access data extraction sheet at http://osf.io/h8xrs.

## Discussion

Our scoping review aimed to map global research on the application of exercise snacks. Overall, 45 eligible publications were identified, resulting from 33 original research studies conducted across eight high-income countries. Study designs utilized in included trials spanned the clinical trials umbrella definition (e.g., mechanistic, exploratory/developmental/pilot/feasibility, other interventional or behavioral), with just over half the included studies described as pilot or feasibility studies by their authors. The range of study designs, coupled with the dominance of small-scale randomized controlled trials, crossover trials, and single group studies, reflects the relative infancy of exercise snack as a research field, where most of the studies conducted to date could be classified as efficacy or proof-of-concept trials [102].

The number of publications on exercise snacks has been on an upward trajectory from 2017, with only four studies published prior to this date. From our systematic search, it appears the first peer-reviewed research publication to explicitly use the concept snacks of exercise was conducted in 2006 [61]. In this study, the impact of four, 10-minute exercise snacks (brisk walking) on blood pressure in hypertensive adults, was compared with moderate-intensity continuous walking and a control condition. While not acknowledged at the time, it is likely this 2006 publication partly inspired the 2007 online Newsweek article by Dr Howard Hartley, entitled “An ‘Exercise Snack’ Plan” [103], which has previously been credited as the first use of the exercise snacks concept (e.g [25, 42]). The last research paper prior to the publication upsurge was by Francois et al. [62], which explored the role of exercise snacks before meals on glycemic control in adults with prediabetes or type 2 diabetes. To date, individuals with a form of diabetes (e.g., pre-diabetes, type 1 diabetes or type 2 diabetes) are the most studied clinical population within exercise snacks research, with four studies (one involving adolescents with type 1 diabetes) included in our review. This is, however, much lower than the number of studies where participants were described as ‘healthy’ (55% of our included studies). Given the nascent nature of the field however, this finding is unsurprising and likely reflects the recognized pathway of clinical trials, whereby treatments are first conducted in healthy individuals, followed by clinical groups only if successful and safe.

The median sample size of our included studies was 24 participants and only 33% of studies provided an *a*-priori sample-size estimation. At odds with other sport and exercise sciences research, where females are typically under-represented [104], all 33 studies included female participants. This is encouraging and, where appropriate, should be maintained in future research. It should, however, be highlighted only a quarter of our included studies reported details on female participants’ menstrual cycle status, where relevant. Given some of the postulated fluctuations in, for example, insulin sensitivity, secretion and glucose during the menstrual cycle [105, 106], this could be an important methodological consideration that should be acknowledged [107] and/or controlled for in future studies [108]. In terms of the global picture, exercise snack research, to date, appears to be concentrated in a small number of high-income countries. Further, the ethnicity of participants was only reported in 27% of our included studies, which negates our ability to conduct cross-cultural comparisons on how exercise snacks can be operationalized. Future research in the area should therefore consider these limitations and take pro-active steps to facilitate representativeness in exercise research, in recruitment strategies and reporting of participants’ characteristics.

Our study concept (i.e., exercise definition) was described by original study authors in a variety of ways. The term exercise snacks was used most frequently (60% of included studies) and represented protocols based on body-weight exercises, body-weight exercises and resistance bands, stair climbing and walking. Interestingly, seven of our included studies utilized cycling protocols, yet only one directly referenced the snack element, via the term sprint snacks [23]. In the only study to describe strength snacks, resistance exercise was performed on leg extension and chest press machines [71]. Other included studies met our definition of exercise snacks (i.e., a structured bout of intense exercise dispersed across the day) yet did not explicitly refer to their protocol as exercise snacks. For example, Bailey et al. [45] prescribed fragmented high-intensity activity breaks as a means of breaking up sedentary time in inactive adults, and Ho et al. [70] and Wun et al. [78] described their protocol as brief, maximal efforts dispersed across the day. This was also largely the case in the small number of studies involving children and adolescents. Here, only one publication defined their protocol as exercise snacks [79], with the remaining two describing their interventions as either high-intensity activity breaks [67] or short bursts of activity interspersed across the school day [60].

While all included studies share the commonality of incorporating brief bouts of purposeful exercise, dispersed throughout the day, it is evident that the exercise snack concept is already being used to refer to different exercise prescriptions in the literature. To ensure future syntheses on the topic can be pooled from appropriate studies, we propose the following. Firstly, we advocate for the consistent use of exercise snacks as a concept, which at this stage could be used as an umbrella term to describe all protocols whereby short, purposeful structured exercise (as opposed to lifestyle physical activity) is dispersed throughout the day. The concept of exercise snacks could replace and/or be used alongside exercise prescription names such as minimal-dose resistance training [3] and fragmented/ dispersed/ accumulated exercise models. This would ensure all applicable concepts are recognized and grouped appropriately in future data synthesizes, and could simplify the concept by minimizing scientific jargon and technical language. To facilitate this further, we also recommend that future studies report sufficient detail on their exercise prescription model that could allow replication. This could aid the creation of sub-categories on an exercise snacks continuum, such as those already described as sprint snacks, strength snacks etc. In recognition of the important role that choice, variety and autonomy play in the psychological and behavioral impact of exercise behaviors [109, 110] we do not advocate for the creation of standardized definition/prescription of exercise snacks, which would not allow for flexibility in exercise programming principles and individual choice. While we recognize the lack of standardized definition may hinder future efforts to synthesize data using statistical methods, we hope that our call to provide detailed descriptions of the exercise prescriptions will alleviate this somewhat. By providing greater flexibility in how the exercise snacks concept is operationalized, we hope this will also facilitate the continuation of studies across a variety of contexts, and further widen the practical application of exercise snacks in daily life settings where appropriate.

To contrast the treatment effects of exercise snack protocols, a variety of comparators were employed, which reflects the differing study designs (i.e., Acute vs. Chronic), study context (laboratory, university, daily life, home or school) and the primary aims and outcomes of the original research trials. The most frequently adopted comparators were non-exercise controls, moderate-intensity continuous exercise and prolonged sitting. Four of the 21 Chronic studies did not have a comparator arm, which has methodological limitations in inferring causal efficacy/effectiveness. In terms of intervention length, most chronic trials were relatively short (median duration of 7 weeks), with only 8/21 chronic training studies conducted for 12 weeks or longer. Of these, six were published in the three years immediately preceding this review [80, 83, 90, 92, 94, 98], which reflects the rapidly increasing pace of exercise snacks research. As the field continues to grow, we recommend trials of longer duration are conducted, with longer-term follow-up where possible. Across all 33 included studies, the mean study completion was very high (93% of participants), which is encouraging. Exercise snack duration, number of snacks performed per day, daily exercise snack duration, rest periods between snacks and the number of days per week snacks were performed varied widely, which further highlights heterogeneity in the way in which exercise snacks are being prescribed with regards to exercise programming principles.

For exercise intensity, 82% of studies provided information on the prescribed intensity of exercise snacks. Intensity prescription method was well reported with was asking participants to complete ‘as many repetitions as possible with appropriate technique/safely’ (*n* = 7) the most common method. Such detail on exercise intensity prescription is reassuring as intensity is very often the essential exercise programming principle [111] and monitoring intensity can provide an objective measure of the extent to which participants complied with the prescribed exercise dose [112]. Nevertheless, ten studies did not provide information on exercise intensity and crucially only 49% provided data of reported exercise intensity, with the mean ± standard deviation of exercise intensity measures being the sole method of reporting. Despite the concerns raised in our introduction on the usefulness of heart rate and RPE for measuring exercise snack intensity, the data extracted from studies illustrated a relatively intense exercise dose. For example, a mean exercise snack heart rate of 76.9% HR_max_ is close to the recommended threshold for high-intensity of 80% HR_max_ [113], and a RPE 5.2 AU equates to ‘hard’ on the CR10 scale. We acknowledge that such reconciliation of exercise intensity data across different exercise modes and protocols is done with caution, especially given the brevity of exercise snack bouts. However, the heart rate and particularly RPE data are encouraging, not only when considering the mediating role exercise intensity plays for training-induced physiological adaptation [114], but also as potential measures for prescribing and reporting the intensity of brief, discrete exercise bouts (i.e., exercise snacks).

While the extracted exercise intensity data provide an overview of exercise snack intensity, it cannot show fidelity of the trial or intervention (i.e., the extent to which it was delivered as intended across all participants [115]). In chronic training studies, for example, repeated exercise snack sessions performed across an intervention period will give rise to between- and within-participant variability in the exercise intensity response. As the observed between-participant standard deviation provides no robust information pertaining to the within-participant variability, and overestimates the true between-participant variability, it is not possible to establish whether the content and process of the intervention was consistent throughout the trial [112]. It is therefore unsurprising that the CERT item most often underreported was Item 16b (intervention fidelity), which was adequately reported in only two of 33 studies. To address these methodological issues in future trials, researchers should give due consideration to how they intend to prescribe, monitor and report the intensity of their exercise snack protocols. This is particularly pertinent for studies where the context is inherently unsupervised, such as exercise snack protocols conducted at home or in daily life. One possible solution is using wearable physical activity trackers [116, 117], which can collect the real-time data either remotely or in-person and would allow data to be analyzed using statistical models which can account for between- and within-participant variations (e.g., appropriate linear mixed modelling [112]).

In comparison to common criticisms around trial and exercise reporting quality in sport and exercise sciences (e.g [43, 44]), the study protocols in our review were largely well described and detailed. Indeed, when mapped onto the CERT [44], studies mostly adhered to the 16 reporting items. Along with intervention fidelity (Item 16b), three items were consistently underreported, namely Item 2 (qualification of the exercise provider), Item 6 (motivation strategies), Item 11 (adverse events). Given the often-intense nature of exercise snack protocols and inclusion of clinical populations in trials, it is particularly concerning that more than half of the studies did not report on adverse events (Item 11), even if simply to report that none occurred. Regardless of population, this should be addressed in all future studies to allow the safety of exercise snacks to be robustly examined, ideally via the CONSORT Harms 2022 checklist [118]. Where possible, detail should also be provided on how risk to participants is mitigated in protocols involving trip hazards, such as stair climbing. For example, in the supervised stair snacks study by Rafiei et al. [39], participants classified as young and healthy weight received an instruction to ascend stairs ‘as quickly and safely as possible’. In contrast, participants who were living with overweight or obesity, were asked to ‘climb the stairs at a self-selected challenging pace’, which acknowledged that stair climbing at a sprint pace was not feasible for some participants. Considering other recent reviews have proposed the potential role of exercise snacks in people living with, and beyond, cancer [40], it will be interesting to observe how research develops in other clinical populations over time. Here, however, it may be necessary for the field to diverge slightly for safety and patient care purposes. For example, our review has provided details of largely unsupervised exercise snack protocols (i.e., performed at home or in daily life) in healthy adults and some older adult groups. As the field continues to grow, it is therefore likely that more protocols in healthy populations, and possibly in groups with well-managed clinical conditions, will be performed without direct supervision/observation. This could be facilitated through the provision of wearable technologies, such as physical activity trackers, heart-rate monitors, smart watches, and arm-mounted continuous glucose monitors for individuals with pre-diabetes and/or type 2 diabetes, as a means of remotely monitoring exercise responses. In other clinical populations, (e.g., those with poorly managed/ multiple long-term conditions or relative or absolute contraindications to exercise), however, it may never be appropriate to perform exercise snacks without direct supervision from researchers, exercise practitioners or healthcare professionals.

Across the studies included in our review, 87 distinct physiological, psychological, and behavioral outcome measures were utilized. These individual outcomes were then collapsed into ten outcome categories for the purposes of reporting (inflammatory, appetite, vascular, anthropometric, feasibility, psychological, cardiovascular, movement, muscular, and metabolic). Most studies included more than one outcome measure, and the most frequently reported outcomes were cardiovascular, metabolic, muscular, and psychological measures. In future research it would be valuable to more consistently incorporate psychological outcomes (e.g., self-efficacy, affect) to both better understand the experience of exercise snacks and consider mediators of behavioral adherence over time. In line with scoping review guidance, we have not extracted nor presented results from our included studies, since they have not undergone a process of critical appraisal, nor been analyzed using advanced data synthesis techniques [55]. This is to ensure misplaced conclusions regarding the efficacy and/or effectiveness (or not) of exercise snacks do not occur because of our review.

Collectively, it is evident that exercise snacks are already being conceptualized through a variety of exercise prescriptions and assessed against a wide range of outcomes which span the human health and well-being spectrum. The heterogeneity of the exercise prescriptions and outcome measures adopted in our included studies does pose some interesting considerations on how exercise snacks could be collectively synthesized in the future. Indeed, while scoping reviews are not designed to address questions of feasibility, appropriateness, meaningfulness, or effectiveness [55], if the field continues to advance at the current pace, there will become a point where a systematic review and/or meta-analysis is deemed appropriate. Here, researchers may need to consider whether exercise snacks can exist as a singular concept, or whether a continuum-based model, similar to those recently observed for HIIT, for example [119], would be more appropriate. Further, while the grouping of diverse populations and study types is appropriate for a scoping review, this approach would not be suitable for reviews aiming to synthesise data from discrete populations, study designs or outcomes. In this eventuality, researchers may wish to focus on a specific group (i.e., youth, adult or clinical populations) and differentiate by study type (i.e., Acute or Chronic studies) and study outcomes where appropriate, to allow valid comparisons to be made. The terminology and reporting guidance we have provided should assist with this, as it is possible that different exercise snack protocols may give rise to different physiological, psychological, and behavioral stimuli and subsequent adaptations, which will require careful consideration in future studies and syntheses.

From our review, it is evident most of the research to date has focused on adult and older populations, with the potential application in children and adolescents still under-researched. More studies in school-age participants therefore represents a potential avenue for future research. Indeed, the dispersed nature of exercise snacks may align well with protocols based around physical activity breaks in school settings [120, 121], provided the exercise intensity is sufficient and the protocol itself well described. Of note, many studies which did not meet these criteria were excluded during the screening process of our review. Considering studies which utilized exercise snacks as a means of breaking up sedentary behavior, a valuable context and population for future studies from a public and occupational health perspective are workplaces and individuals with sedentary-based occupations. Four of our included studies have begun to explore this context, via supervised snack protocols conducted in university premises [23, 39, 94, 96], while only one study to date has utilized an unsupervised workday protocol [89]. It could therefore be postulated that a balance is needed between protocols which have the possibility for scale-up for public health benefit (i.e., able to be conducted without supervision), yet can also be conducted safely and with high fidelity.

Given the infancy of exercise snacks field, it is unsurprising that laboratory-based studies still represent the largest proportion of study contexts in our review and study sample sizes are still relatively small. To alleviate concerns over non-replicable science, future studies should pre-register their protocols, conduct *a*-priori sample size estimations (procedures poorly conducted by studies included in our review), and avoid combining many dependent variables with small study samples [122]. Trial and exercise reporting in future studies should continue to follow guidance such as the CERT and CONSORT Harms template [44, 118] and utilize appropriate equipment and techniques to adequately evaluate the exercise treatment fidelity.

The reporting of CERT items from our included studies is a novel and key strength of our review, highlighting items which were consistently underreported. Nonetheless, while our scoping review has comprehensively identified and mapped the available evidence on exercise snacks, it is not without limitations. First, we were only able to include studies published in the English language, therefore trials published in other languages would have been missed. This limitation may be reflected by our inclusion of only five studies conducted in countries where English is not the first language. Second, while we sought to include studies involving children and adolescents and clinical groups, these populations were represented by a relatively small number of studies in our review. Finally, as a result of the included studies, rather than the scoping review process itself, it was apparent that some important trial and exercise reporting practices were underreported across all studies, namely measures and explanations of intervention fidelity and reporting of adverse events. For those involved in the research, science, and practice of exercise snacks prescription and promotion, monitoring the safety, appropriateness, and achievement of such prescriptions is paramount, and should be a key focus of future research and practice.

## Conclusions

From our review, it is evident that exercise snacks are already being applied through an array of concepts and contexts on a wide range of outcome measures related to human health and well-being, albeit largely in relatively small samples of adult and older adults. If the field continues to grow, researchers should strive to recruit larger and more diverse samples into their studies and continue to prescribe and report detailed exercise snacking protocols which are suitable for their study populations, and replicable by other research groups.

## Electronic Supplementary Material

Below is the link to the electronic supplementary material.

Supplementary Material 1

Supplementary Material 2

Supplementary Material 3

## Data Availability

The datasets generated during and/or analysed during the current study are available in the Open Science Framework repository, http://osf.io/h8xrs, and in Supplementary File 3.

## Abbreviations

ACSM: American College of Sports Medicine
AU: Arbitrary Units
BASES: British Association of Sport and Exercise Sciences
CERT: Consensus on Exercise Reporting Template
CONSORT: CONsolidated Standards Of Reporting Trials
ECSS: European College of Sport Science
FITT-VP: Frequency, Intensity, Time, Type, Volume, and Progression
HIIT: High-Intensity Interval Training
%HR_max_: Percentage of maximal heart rate
JBI: Joanna Briggs Institute
MET: Metabolic Equivalent of Task
PCC: Population, Concept [including Outcome], and Context
PRESS: Peer Review of Electronic Search Strategies
RPE: Rating of Perceived Exertion
SD: Standard Deviation
SIT: Sprint Interval Training
TiDIER: Template for Intervention Description and Replication
UK: United Kingdom
USA: United States of America
VO_2max_: Maximal Oxygen Consumption
VILPA: Vigorous Intermittent Lifestyle Physical Activity

## Declarations

### Scoping Review registration

Our scoping review protocol was registered on the Open Science Framework (http://osf.io/h8xrs).
