# Supplementary material for: Application of Exercise Snacks across Youth, Adult and Clinical Populations: A Scoping Review
Source: Sports Med Open. 2025 Mar 18;11:27. doi: 10.1186/s40798-025-00829-6 (PMC11920532; doi:10.1186/s40798-025-00829-6)
Supplement: Supplementary file 2 — Supplementary Material 2 [file 40798_2025_829_MOESM2_ESM.pdf]

Sports Medicine Open

Application of Exercise Snacks across Youth, Adult and Clinical populations: A Scoping Review.

Kathryn L Weston[1*], Jonathan P Little[2], Matthew Weston[3,4,5], Sara McCreary[2], Vanessa Kitchin[2], Amrit Gill[2], Ailsa Niven[4,6], Melitta A McNarry[3$] and Kelly A Mackintosh[3$]

[1]Department of Psychological Sciences and Health, University of Strathclyde, Glasgow, UK.

[2]School of Health and Exercise Sciences, University of British Columbia, Okanagan, Kelowna, British Columbia, Canada.

[3]Applied Sports, Technology, Exercise and Medicine (A-STEM) Research Centre, Swansea University, UK.

[4]Institute for Sport, Physical Education and Health Science, Moray House School of Education and Sport, University of Edinburgh, Edinburgh, UK.

[5]Institute of Sport, Manchester Metropolitan University, Manchester, UK.

[6]Physical Activity for Health Research Centre (PAHRC), University of Edinburgh, Edinburgh, UK.

$ Joint last authors

*Corresponding author: Dr Kathryn L Weston. Email: kathryn.weston@strath.ac.uk

Database: Ovid MEDLINE(R) and Epub Ahead of Print, In-Process, In-Data-Review & Other Non-Indexed Citations, Daily and Versions <1946 to November 24, 2024>
Search Strategy:

1 exercise/ or physical conditioning, human/ or circuit-based exercise/ or running/ or Stair Climbing/ or bicycling/ or exercise therapy/
2 high-intensity interval training/ or muscle fatigue/ or physical exertion/ or plyometric exercise/
3 resistance training/ or weight lifting/
4 ((high or vigorous) adj2 intens* adj3 (bout? or burst? or snack? or snacking or breaks)).tw.
5 ((((high or vigorous) adj2 intens*) and (training or activit*)) adj3 (brief or interval? or intermittent or sporadic or cycle? or hourly or short or dispersed)).tw.
6 ((sprint? or circuit-based or all-out or plyometric* or resistance) adj2 (bout? or burst? or brief or snack? or snacking or breaks)).tw.
7 ((sprint or circuit-based or all-out or plyometric or resistance) adj2 (training or activit*) adj3 (brief or interval? or intermittent or sporadic or cycle? or hourly or short or dispersed)).tw.
8 ((extreme or strength or strenuous) adj2 (training or activit*) adj3 (brief or interval? or intermittent or sporadic or cycle? or hourly or short or dispersed)).tw.
9 ((exercise or bicycle or cycle or weight-lifting) adj2 (bout? or burst? or snack? or snacking)).tw.
10 ((extreme or strength or strenuous) adj2 (bout? or burst? or snack? or snacking)).tw.
11 (((exercise or bicycle or weight-lifting or running) adj2 (brief or interval? or intermittent or sporadic or cycle? or hourly or short or dispersed)) and (high or vigorous or extreme or strenuous or intens*)).tw.
12 (bout? or burst? or snack? or snacking or accumulated or dispersed).m_titl.
13 or/1-3
14 12 and 13
15 or/4-11
16 14 or 15
17 limit 16 to (english language and humans)

Database: Embase <1974 to 2024 November 24>
Search Strategy:

1 exercise/ or circuit training/ or running/ or Stair Climbing/ or cycling/
2 high intensity exercise/ or exercise intensity/ or anaerobic exercise/ or plyometrics/ or muscle fatigue/
3 resistance training/ or weight lifting/
4 ((high or vigorous) adj2 intens* adj3 (bout? or burst? or snack? or snacking or breaks)).tw.
5 ((((high or vigorous) adj2 intens*) and (training or activit*)) adj3 (brief or interval? or intermittent or sporadic or cycle? or hourly or short or dispersed)).tw.
6 ((sprint? or circuit-based or all-out or plyometric* or resistance) adj2 (bout? or burst? or brief or snack? or snacking or breaks)).tw.

7 ((sprint or circuit-based or all-out or plyometric or resistance) adj2 (training or activit*) adj3 (brief or interval? or intermittent or sporadic or cycle? or hourly or short or dispersed)).tw.
8 ((extreme or strength or strenuous) adj2 (training or activit*) adj3 (brief or interval? or intermittent or sporadic or cycle? or hourly or short or dispersed)).tw.
9 ((exercise or bicycle or cycle or weight-lifting) adj2 (bout? or burst? or snack? or snacking)).tw.
10 ((extreme or strength or strenuous) adj2 (bout? or burst? or snack? or snacking)).tw.
11 (((exercise or bicycle or weight-lifting or running) adj2 (brief or interval? or intermittent or sporadic or cycle? or hourly or short or dispersed)) and (high or vigorous or extreme or strenuous or intens*)).tw.
12 (bout? or burst? or snack? or snacking or accumulated or dispersed).m_titl.
13 or/1-3
14 12 and 13
15 or/4-11
16 14 or 15
17 limit 16 to (english language and humans)

Database: Web of Science Core Collection (University of British Columbia)
Editions =Science Citation Index Expanded (SCI-EXPANDED)--1900-present; Social Sciences Citation Index
(SSCI)--1956-present; Arts & Humanities Citation Index (AHCI)--1975-present; Conference Proceedings Citation Index – Science (CPCI-S)--1990-present

Results from Web of Science Core Collection run November 24, 2024:

(((high or vigorous) NEAR/2 (intens*)) NEAR/3 (bout? or burst? or snack? or snacking or breaks)) or (((high or vigorous) NEAR/1 (training or exercise)) NEAR/2 (brief or interval? or intermittent or sporadic or cycle? or hourly or short or dispersed)) or ((sprint? or circuit-based or all-out or plyometric* or resistance) NEAR/2 (bout? or burst? or brief or snack? or snacking or breaks)) or ((sprint or circuit-based or all-out or plyometric or resistance) NEAR/2 (training or activit*) NEAR/3 (brief or interval? or intermittent or sporadic or cycle? or hourly or short or dispersed)) or ((extreme or strength or strenuous) NEAR/2 (training or activit*) NEAR/3 (brief or interval? or intermittent or sporadic or cycle? or hourly or short or dispersed)) or ((exercise or bicycle or cycle or weight-lifting) NEAR/2 (bout? or burst? or snack? or snacking)) or ((extreme or strength or strenuous) NEAR/2 (bout? or burst? or snack? or snacking)) or TS=((((exercise or bicycle or weight-lifting or running) NEAR/2 (brief or interval? or intermittent or sporadic or cycle? or hourly or short or dispersed)) and (high or vigorous or extreme or strenuous or intens*))) and English (Languages)

Database - CINAHL Complete
Interface - EBSCOhost Research Databases
Search Screen - Advanced Search

S1	(MH "Exercise+") OR (MH "High-Intensity Interval Training") OR (MH "Anaerobic Exercises") OR (MH "Plyometrics") OR (MH "Physical Activity") OR (MH "Resistance Training") OR (MH "Anaerobic Threshold")
S2	TI ((bout* or burst* or snack* or snacking or accumulated or dispersed)) OR AB ((bout* or burst* or snack* or snacking or accumulated or dispersed))
S3	TI ((((high or vigorous) N2 intens*) and (training or activit*)) N2 (brief or interval* or intermittent or sporadic or cycle* or hourly or short or dispersed))) OR AB ((((high or vigorous) N2 intens*) and (training or activit*)) N2 (brief or interval* or intermittent or sporadic or cycle* or hourly or short or dispersed)))
S4	TI (((high or vigorous) N2 intens* N3 (bout* or burst* or snack* or snacking or breaks))) OR AB (((high or vigorous) N2 intens* N3 (bout* or burst* or snack* or snacking or breaks)))
S5	TI ((sprint* or circuit-based or all-out or plyometric* or resistance) N2 (bout* or burst* or brief or snack* or snacking or breaks)) OR AB ((sprint* or circuit-based or all-out or plyometric* or resistance) N2 (bout* or burst* or brief or snack* or snacking or breaks))
S6	TI ((sprint or circuit-based or all-out or plyometric or resistance) N2 (training or activit*) N3 (brief or interval* or intermittent or sporadic or cycle* or hourly or short or dispersed))) OR AB (((sprint or circuit-based or all-out or plyometric or resistance) N2 (training or activit*) N3 (brief or interval* or intermittent or sporadic or cycle* or hourly or short or dispersed)))
S7	TI (((extreme or strength or strenuous) N2 (training or activit*) N3 (brief or interval* or intermittent or sporadic or cycle* or hourly or short or dispersed))) OR AB (((extreme or strength or strenuous) N2 (training or activit*) N3 (brief or interval* or intermittent or sporadic or cycle* or hourly or short or dispersed)))

S8	TI (((exercise or bicycle or cycle or weight-lifting) N2 (bout* or burst* or snack* or snacking))) OR AB (((exercise or bicycle or cycle or weight-lifting) N2 (bout* or burst* or snack* or snacking)))
S9	TI (((extreme or strength or strenuous) N2 (bout* or burst* or snack* or snacking))) OR AB (((extreme or strength or strenuous) N2 (bout* or burst* or snack* or snacking)))
S10	TI ((((exercise or bicycle or weight-lifting or running) N2 (brief or interval* or intermittent or sporadic or cycle* or hourly or short or dispersed)) and (high or vigorous or extreme or strenuous or intens*))) OR AB ((((exercise or bicycle or weight-lifting or running) N2 (brief or interval* or intermittent or sporadic or cycle* or hourly or short or dispersed)) and (high or vigorous or extreme or strenuous or intens*)))
S11	S1 AND S2
S12	S3 OR S4 OR S5 OR S6 OR S7 OR S8 OR S9 OR S10 OR S11
S13	S11 OR S12
S13	Narrow by Language: - English

Database - SPORTDiscus
Interface - EBSCOhost Research Databases

S13	S3 OR S4 OR S5 OR S6 OR S7 OR S8 OR S9 OR S10 OR S11<br>**Narrow by Language:** - english
S12	S3 OR S4 OR S5 OR S6 OR S7 OR S8 OR S9 OR S10 OR S11

S11	S1 AND S2
S10	TI (((exercise or bicycle or weight-lifting or running) N2 (brief or interval* or intermittent or sporadic or cycle* or hourly or short or dispersed)) and (high or vigorous or extreme or strenuous or intens*))
S9	TI ((extreme or strength or strenuous) N2 (bout* or burst* or snack* or snacking))
S8	TI ((exercise or bicycle or cycle or weight-lifting) N2 (bout* or burst* or snack* or snacking))
S7	TI ((extreme or strength or strenuous) N2 (training or activit*) N3 (brief or interval* or intermittent or sporadic or cycle* or hourly or short or dispersed))
S6	TI ((sprint or circuit-based or all-out or plyometric or resistance) N2 (training or activit*) N3 (brief or interval* or intermittent or sporadic or cycle* or hourly or short or dispersed))
S5	TI ((sprint* or circuit-based or all-out or plyometric* or resistance) N2 (bout* or burst* or brief or snack* or snacking or breaks))
S4	TI ((high or vigorous) N2 intens* N3 (bout* or burst* or snack* or snacking or breaks))
S3	TI (((high or vigorous) N2 intens*) and (training or activit*)) N2 (brief or interval* or intermittent or sporadic or cycle* or hourly or short or dispersed))
S2	TI bout* or burst* or snack* or snacking or accumulated or dispersed
S1	((DE "EXERCISE" OR DE "ANAEROBIC exercises" OR DE "CIRCUIT training" OR DE "PLYOMETRICS" OR DE "RUNNING" OR DE "TREADMILL exercise" OR DE "ELLIPTICAL trainers" OR DE "PHYSICAL training & conditioning") OR (DE "INTERVAL training")) AND (DE "HIGH-intensity interval training" OR DE "INTERVAL training")

Database: Scopus

University of British Columbia Library Institutional Access

TITLE-ABS-KEY (((high or vigorous) W/2 (intens*)) W/3 (bout? or burst? or snack? or snacking or breaks))
OR
(((high or vigorous) W/1 (training or exercise)) W/2 (brief or interval? or intermittent or sporadic or cycle? or hourly or short or dispersed))
OR
((sprint? or circuit-based or all-out or plyometric* or resistance) W/2 (bout? or burst? or brief or snack? or snacking or breaks))
OR
((sprint or circuit-based or all-out or plyometric or resistance) W/2 (training or activit*) W/3 (brief or interval? or intermittent or sporadic or cycle? or hourly or short or dispersed))
OR
((extreme or strength or strenuous) W/2 (training or activit*) W/3 (brief or interval? or intermittent or sporadic or cycle? or hourly or short or dispersed))
OR
((exercise or bicycle or cycle or weight-lifting) W/2 (bout? or burst? or snack? or snacking))
OR
((extreme or strength or strenuous) W/2 (bout? or burst? or snack? or snacking))
AND
(LIMIT-TO (("Human")) AND (LIMIT-TO (LANGUAGE , "English"))

Grey Literature Searches

PolicyCommons
"exercise snack*" OR "exercise bout*" OR "exercise break*" OR "exercise burst*" OR "accumulated exercise" OR "physical activity bout*" OR "physical activity break*" OR "physical activity burst*" OR "accumulated physical activity" OR "physical activity snack*"

ProQuest Dissertations & Theses Global
ti("exercise snack*" OR "exercise bout*" OR "exercise break*" OR "exercise burst*" OR "accumulated exercise" OR "physical activity bout*" OR "physical activity break*" OR "physical activity burst*" OR "accumulated physical activity" OR "physical activity snack*")
